# Incorporating cyclone risk in the design of marine protected and conserved areas as an ecosystem‐based adaptation approach

**DOI:** 10.1111/cobi.14437

**Published:** 2025-02-25

**Authors:** Alyssa L. Giffin, Vivitskaia J. D. Tulloch, Dominic A. Andradi‐Brown, Rod M. Connolly, Unaisi Malani‐Tagicakibau, Francis Areki, Christopher J. Brown

**Affiliations:** ^1^ Coastal and Marine Research Centre, Australian Rivers Institute, School of Environment and Science Griffith University Gold Coast Queensland Australia; ^2^ Australian Rivers Institute Griffith University Nathan Queensland Australia; ^3^ Department of Forest and Conservation Science University of British Columbia Vancouver British Columbia Canada; ^4^ Ocean Conservation World Wildlife Fund Washington, DC USA; ^5^ World Wildlife Fund‐Pacific Suva Fiji; ^6^ Institute for Marine and Antarctic Studies University of Tasmania Hobart Tasmania Australia

**Keywords:** climate change, conservation planning, extreme events, marine spatial planning, MarProb, Marxan, resilience, threat mapping, cambio climático, evento extremo, planeación de la conservación, planeación espacial marina, 海洋空间规划, 保护规划, 气候变化, 极端事件, Marxan软件, MarProb工具, 恢复力, 威胁绘图

## Abstract

Marine protected and conserved areas (MPCAs) are promoted as an ecosystem‐based adaptation (EbA) approach to increase community and ecosystem resilience to climate change. However, traditional approaches to MPCA design typically do not consider climate risk or habitat condition under a climate threat. We used the Great Sea Reef (GSR) in Fiji as a case study to develop a land–sea prioritization framework that links modeled sediment runoff from rainfall during extreme cyclone events to the probability of coral reefs being in good condition. We incorporated this information in an MPCA prioritization scenario intending to achieve 90% certainty of good‐condition coral cover under cyclone risk while minimizing cost to fishers and meeting ecosystem conservation targets. We explored the trade‐offs between sites selected for protection, the relative opportunity cost to fishers, and the representation of conservation feature targets between the MPCA scenario that included cyclone risk and a baseline scenario that did not. The cyclone risk scenario's best solution required larger areas of protection (5% more GSR area) than the baseline scenario and additional protection in areas with moderate to high probability of good‐condition coral cover. Some areas prioritized for protection in the cyclone risk scenario had relatively high turbidity. Large sections around Vanua Levu were consistently selected for protection across both prioritization scenarios due to high concentrations of all ecosystem conservation features, particularly sea turtle feeding grounds. Overall, the cyclone risk MPCA design had a higher fisher opportunity cost but protected a larger amount of ecosystem conservation features and buffered against habitat condition uncertainty. We explored the potential outcomes of expanding on threat‐avoidance and cost‐effective conservation prioritization by including habitat responses to threats in the prioritization process. Our findings can inform MPCA design during EbA planning in regions at risk from climate change.

## Abstract (es)

Incorporación del riesgo de ciclones en el diseño de las áreas marinas protegidas y conservadas como una estrategia de adaptación basada en el ecosistema

**Resumen:**

Las áreas marinas protegidas y conservadas (AMPC) se promueven como un enfoque de adaptación basado en los ecosistemas (ABE) para aumentar la resiliencia de las comunidades y los ecosistemas al cambio climático. Sin embargo, los enfoques tradicionales de diseño de las AMPC no suelen considerar el riesgo climático ni el estado del hábitat en caso de amenaza climática. Utilizamos el Gran Arrecife Marino (GSR) de Fiyi como estudio de caso para desarrollar un marco de priorización tierra‐mar que vincula la escorrentía de sedimentos modelada a partir de las precipitaciones durante eventos ciclónicos extremos con la probabilidad de que los arrecifes de coral estén en buenas condiciones. Incorporamos esta información en un escenario de priorización AMPC con el objetivo de alcanzar un 90% de certeza de cobertura de coral en buen estado bajo riesgo de ciclón, minimizando al mismo tiempo el coste para los pescadores y cumpliendo los objetivos de conservación del ecosistema. Exploramos las compensaciones entre los lugares seleccionados para la protección, el coste de oportunidad relativo para los pescadores y la representación de los objetivos de conservación entre el escenario AMPC que incluía el riesgo de ciclón y un escenario de referencia que no lo incluía. La mejor solución del escenario de riesgo de ciclones requería zonas de protección más amplias (un 5% más de superficie de GSR) que el escenario de referencia y protección adicional en zonas con una probabilidad de moderada a alta de cobertura de coral en buen estado. Algunas zonas prioritarias para la protección en el escenario de riesgo de ciclón tenían una turbidez relativamente alta. En ambos escenarios de priorización se seleccionaron para su protección grandes secciones alrededor de Vanua Levu debido a las altas concentraciones de todos los elementos de conservación del ecosistema, en particular las zonas de alimentación de las tortugas marinas. En general, el diseño del AMPC con riesgo de ciclón tuvo un mayor costo de oportunidad para los pescadores, pero protegió una mayor cantidad de elementos de conservación del ecosistema y amortiguó la incertidumbre sobre el estado del hábitat. Exploramos los posibles resultados de la ampliación de la prevención de amenazas y la priorización de la conservación rentable mediante la inclusión de las respuestas del hábitat a las amenazas en el proceso de priorización. Nuestras conclusiones pueden servir de base para el diseño de AMPC durante la planificación de ABE en regiones amenazadas por el cambio climático.

## 将气旋风险纳入海洋保护区设计以作为一种基于生态系统的适应方法

【摘要】 海洋保护区(marine protected and conserved areas, MPCA)这种基于生态系统的适应方法已得到推广, 旨在提高社区和生态系统应对气候变化的恢复力。然而, 传统的MPCA设计方法通常不考虑气候风险或气候威胁下的栖息地情况。本研究以斐济大海礁为案例, 开发了一个海陆优先保护框架, 整合了极端气旋事件期间降雨产生的沉积物径流模拟与珊瑚礁是否处于良好状态的概率。我们将这一信息纳入一个MPCA优先保护方案, 以在气旋风险下确保90%的珊瑚覆盖保持良好状态, 同时最大限度地降低渔民的成本并实现生态系统保护目标。研究探究了包括气旋风险的MPCA方案与不包括气旋风险的基线方案之间, 在保护位点选择、渔民的相对机会成本, 以及保护特征目标代表性方面的利弊权衡。结果发现, 与基线方案相比, 气旋风险方案的最佳解决方案需要更大的保护面积(多保护大海礁面积的5%), 还需在有中高概率维持珊瑚覆盖良好状态的区域提供额外保护。气旋风险方案中优先保护的一些区域浊度相对较高。瓦努阿莱武岛周围的大片区域在两种优先保护方案中都被一致选为保护区, 因为这些区域集中了所有生态系统保护特征, 尤其是包括了海龟觅食地。总体而言, 气旋风险下MPCA设计的渔民机会成本较高, 但保护了更多的生态系统保护特征, 并缓冲了栖息地条件的不确定性。本研究探究了通过将栖息地对威胁的响应纳入优先保护规划过程来扩大威胁规避和成本效益保护优先规划的潜在结果。该研究结果可为气候变化风险区域基于生态系统适应的规划中的MPCA设计提供参考。**【翻译:胡怡思;审校:聂永刚】**

## INTRODUCTION

Millions of people living in coastal communities worldwide are already experiencing the adverse effects of climate change, particularly those who depend on coastal ecosystems for their food and livelihoods (Savo et al., 2016; Thomas et al., 2019). In these coastal areas, coral reef ecosystems are especially important for sustaining biodiversity and fisheries (Hughes et al., 2017; Teh et al., 2013). Unfortunately, coral reefs in these coastal areas are particularly at risk of the direct and indirect threats of extreme cyclone events that are expected to increase in frequency with climate change in many regions (Bloemendaal et al., 2022; Krauss & Osland, 2020). In tropical regions, cyclones have caused widespread damage to coral reefs through damaging winds and waves (Cheal et al., 2017; Mangubhai, 2016). In addition to the direct damage caused by cyclones, increased rainfall during cyclone events can lead to excessive sediment runoff in coastal catchments that can smother and directly harm coral or reduce light penetration needed for coral growth and survival (Dunstan et al., 2018; Fabricius et al., 2008; Wenger et al., 2016). Changes in coral cover, complexity, and health can affect the composition of fish assemblages and reduce fisheries biomass (Rogers et al., 2014). Moreover, increasing turbidity levels on coral reefs may directly reduce fish species richness, biomass, or settlement success of some fish species (Bejarano & Appeldoorn, 2013; Moustaka et al., 2018; Wenger et al., 2011). Management decisions for Pacific Island Countries and Territories (PICTs) that rely heavily on these coastal ecosystems for subsistence fisheries and livelihoods must include adaptation options for communities that incorporate the possible risk of more extreme cyclonic events under climate change.

Ecosystem‐based adaptation (EbA) is an adaptation approach that focuses on integrating biodiversity conservation and the protection of ecosystem services to increase community resilience to climate change events (Giffin et al., 2020). A key tool for implementing EbA is marine protected and conserved areas (MPCAs), which support biodiversity while also maintaining livelihoods and food security and increasing the capacity of communities to adapt to climate change events (Giffin et al., 2020). Widely promoted as an ecosystem‐based management tool, MPCAs are implemented to address direct threats to coastal ecosystems, protect biodiversity, and sustain fisheries (Gaines et al., 2010; Halpern et al., 2010). Effectively managed MPCAs can deliver benefits for fisheries as well as ecological benefits, such as increased fish biomass and species density and diversity within their boundaries (Lester & Halpern, 2008; Russ et al., 2003). Traditional approaches to the marine spatial planning or conservation planning of MPCAs use generic strategies or design principles to ensure that biodiversity and habitat targets are met in places of reduced cost to fishers (Ban et al., 2009; Gaines et al., 2010). These approaches assume that areas selected for protection will retain their ecological features but often ignore the potential of external stressors that can continue to degrade habitats and compromise the ability of MPCAs to provide their intended services to local communities (Game, Watts, et al., 2008; Klein et al., 2013). Consequently, when MPCAs are used as an EbA action, they could fall short of their biodiversity and fisheries objectives if designed without considering the risk of habitat destruction from climatic events, such as cyclones.

Incorporating the risk of habitat degradation from environmental stressors when planning MPCAs as an EbA approach poses new challenges to traditional conservation and threat‐based planning approaches. Commonly, including threats in planning prioritizations involves selecting areas for action that avoid high threats (Ban et al., 2012; Tulloch et al., 2015). Prioritization tools now exist that can account for the risk of habitat degradation by incorporating the probability of impact or ecological response from such events in the marine spatial planning process (Watts et al., 2021). Planners can use these tools to choose to minimize threat risk by selecting areas for protection with a higher chance of being in good condition under a specific threat (Game, Watts, et al., 2008; Klein et al., 2013; Powers et al., 2017). A growing number of marine spatial planning approaches incorporate climate threats and refugia areas in their design of MPCAs (Jones et al., 2016). Many marine applications focus on the threat of coral bleaching based on historical time‐series analysis or future projections of sea surface temperatures (Ban et al., 2012; Game, Watts, et al., 2008; Levy & Ban, 2013). Comparably few examples exist of MPCA spatial planning that incorporate cyclone risk (but see Game, McDonald‐Madden, et al. [2008]), particularly the indirect threat they may pose to coral reefs due to sediment runoff and degraded water quality. The lack of cyclone risk consideration in conservation planning may largely be due to the high complexity of predicting cyclonic events and the lack of fine‐scale data needed to include their risk in MPCA design at practical management scales (Beyer et al., 2018; Dunstan et al., 2018; Knutson et al., 2010). However, given that many data‐poor regions are experiencing some of the worst impacts of cyclonic events (Moritz et al., 2018; Thomas et al., 2019), spatial planning of MPCAs that uses the best available data on cyclone risk should be explored alongside traditional approaches to reduce vulnerability of communities to climate change and improve socioecological outcomes for a given region.

We developed a land–sea prioritization framework for the implementation of MPCAs as an EbA approach with an overarching objective to reduce community vulnerability to climate change through 3 key socioecological objectives: avoid protecting coral reefs in poor condition as a result of sediment runoff from extreme cyclone events; minimize the opportunity cost of ecosystem protection to fishers; and meet ecosystem and biodiversity conservation targets to help sustain coral reef fisheries. We applied our spatial planning approach in the Great Sea Reef (GSR) in Fiji. We used the best available data on historical cyclones and rainfall, marine and coastal ecosystem extent, and coral reef cover to determine the spatially explicit probability of sediment runoff during extreme cyclone events resulting in low levels of hard coral cover along the GSR. We used this information to compare spatial outcomes between a traditional MPCA planning approach (e.g., meet biodiversity targets while minimizing cost), with an approach that accounts for the condition of coral given sedimentation from extreme cyclone events. Specifically, we sought to answer the following questions: which areas in the GSR are the most at risk of high sediment runoff from extreme cyclone events, which areas in the GSR have the highest probability of coral cover in good condition (i.e., probability of hard coral cover >30%) under sediment runoff levels during extreme cyclone events, how does incorporating cyclone risk in the marine spatial planning process change the priority sites selected for protection compared with planning that does not consider threat risk to habitats, and how does accounting for cyclone risk influence the trade‐offs between opportunity costs to fishers and achieving ecosystem conservation targets?

## METHODS

### Land–sea prioritization framework

We extended previous land–sea planning approaches (Delevaux et al., 2018; Tulloch et al., 2016) to develop a holistic land–sea prioritization framework that links outputs from a sediment runoff model based on rainfall during extreme cyclone events to a plume dispersal model of total suspended sediment (TSS) and modeled the probability of coral cover being in good condition (>30% hard coral cover) based on these TSS outputs on coral reefs. The outputs from the sediment and coral cover probability model were included in a prioritization scenario that accounted for cyclone risk and coral condition certainty in the design of MPCAs as an EbA approach. This scenario was then compared against a baseline prioritization scenario that did not account for cyclone risk and coral degradation to evaluate differences in MPCA network priorities between traditional conservation planning methods and those that accounted for extreme cyclone events under climate change.

### Planning region

**FIGURE 1:**
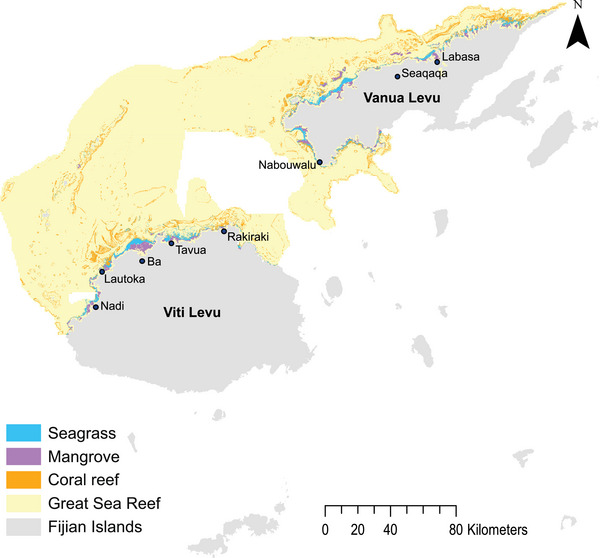
Fijian Islands and ecosystem conservation features in the Great Sea Reef, which was used as a case study to develop a land–sea prioritization framework that links modeled sediment runoff from rainfall during extreme cyclone events to the probability of coral reefs being in good condition. *Coral reef* is defined as coral and algae benthic substrata (Andradi‐Brown et al., 2022), major cities and towns adjacent to the GSR are displayed, and population counts of these locations are used to calculate fisher opportunity loss costs. Sea turtle feeding grounds are not displayed due to data sensitivities.

Our case study encompassed the GSR in Fiji and comprised planning units of 1 × 1 km clipped to the customary *qoliqoli* fishing ground boundaries in the region (Figure 1; Andradi‐Brown et al., 2022). The GSR extends from western Viti Levu to the eastern tip of Vanua Levu (Andradi‐Brown et al., 2022). It consists of a complex network of coral reefs, mangroves, and seagrass beds that support a high diversity of species (Andradi‐Brown et al., 2022). Many coastal communities in the region depend on inshore ecosystems for subsistence fishing and livelihoods (Andradi‐Brown et al., 2022; Thomas et al., 2019). We chose the GSR in Fiji as our case study region because it has experienced the adverse effects of extreme cyclone events, there is a high level of local dependence on coastal and marine ecosystems for subsistence fishing, and there is increasing interest from conservation organizations to implement sustainable ecosystem‐based management and climate adaptation actions (Andradi‐Brown et al., 2022; Giffin et al., 2020; Mangubhai, 2016; Thomas et al., 2019). Our planning region and study design were developed in consultation with conservation practitioners from the World Wildlife Fund (WWF) Pacific and US offices, including conservation managers specifically based in Fiji.

### Spatial planning and prioritization approach

We considered 2 prioritization scenarios for designing MPCAs as an EbA approach in the GSR: baseline and cyclone risk. The baseline scenario represented traditional MPCA planning that seeks to meet biodiversity and ecosystem conservation feature targets while minimizing opportunity costs to fishers. The cyclone risk scenario represented MPCA planning that seeks to meet biodiversity and ecosystem conservation feature targets while minimizing the opportunity cost to fishers and incorporating the probability that coral cover is in good condition (>30% hard coral cover) when exposed to sediment runoff from extreme cyclone events.

For the baseline scenario, we used the traditional version of the conservation planning software Marxan to prioritize areas for protection (Ball et al., 2009). Marxan solves the minimum set problem by finding priority areas for protection that minimize cost, subject to the constraint that representation of conservation target features is achieved (see Table 1 for targets):

(1) $$\mathrm{minimize}=\sum_{i}^{N}c_{i}x_{i},$$

(2) $$\mathrm{subject}\;\mathrm{to}=\sum_{i=1}^{N}a_{ij}x_{i}\geq T_{j}\;\forall j,$$

where *x_i_* is a control variable indicating whether the planning unit (*i* = 1 … *N*) was selected for protection (*x_i_* = 1) or not (*x_i_* = 0) and *c_i_* is the planning unit cost (Equation 1). Equation (2) is the constraint imposed to ensure that the target *T_j_* for all habitats (*j* = 1 … *M*) is achieved, where *a_ij_* is the amount of feature *j* in planning unit *i*. The representation constraint (Equation 2) is implemented through a penalty function in the objective function.

**TABLE 1:** Conservation features, associated targets, and spatial data sources associated with development of the Marxan and MarProb marine protected and conserved area prioritization scenarios.

Conservation feature	Target	Target reference	Spatial data source
Coral reefs	30%	Fijian Government agreed to 30% effective area‐based conservation as part of the 30×30 targets outlined in the Global Biodiversity Framework (CBD, 2022) and a 30% target for marine and coastal areas in the National Biodiversity Strategy and Action Plan for Fiji (Department of Environment, 2020).	Geomorphic maps from Allen Coral Atlas (2020) generated through a hierarchical, object‐based classification approach (see https://allencoralatlas.org/methods/ for more details); *coral reef* defined as the presence of coral and algae benthic substrata (Andradi‐Brown et al., 2022)
Mangroves	30%	Fijian Government agreed to 30% effective area‐based conservation as part of the 30×30 targets outlined in the Global Biodiversity Framework (CBD, 2022) and a 30% target for marine and coastal areas in the National Biodiversity Strategy and Action Plan for Fiji (Department of Environment, 2020).	Atkinson et al. (2016) based on the global mangrove data set from Hamilton and Casey (2016) combined with habitat maps digitized by the Fiji Department of Forestry with satellite imagery
Seagrass	30%	Fijian Government agreed to 30% effective area‐based conservation as part of the 30×30 targets outlined in the Global Biodiversity Framework (CBD, 2022) and a 30% target for marine and coastal areas in the National Biodiversity Strategy and Action Plan for Fiji (Department of Environment, 2020).	Geomorphic maps from Allen Coral Atlas (2020) generated through a hierarchical, object‐based classification approach (see https://allencoralatlas.org/methods/ for details)
Sea turtle feeding grounds	10%	Key endangered species conservation feature that may also be affected by sediment runoff	Ancedotal location data provided by World Wildlife Fund (WWF); digitized approximate locations used

For the cyclone risk scenario, we used the modified version of Marxan, Marxan with Probability (hereafter MarProb), that includes species or threat distribution probabilities. MarProb improves on standard prioritization approaches by accounting for uncertainty (Watts et al., 2021). MarProb solves a minimum set problem similar to traditional Marxan; however, a probabilistic constraint is used instead of the deterministic constraint (Equation 3) and is implemented through a penalty term on the objective:

(3) $$p_{j}\left(x,\;T_{j}\right)\geq P_{j}\forall_{j},\mathrm{where}\;0<p_{ij}<1,$$

where *p_j_* (*x*,*T_j_*) is the probability that feature *j* meets the target (*T_j_*) given protected area system *x*, and *P_j_* is the level of certainty with which one wishes to meet that target (Watts et al., 2021). We assigned a probability to each planning unit (*p_i_*) that corresponds with the estimated probability that hard coral cover will be in good condition after being exposed to the threat of sediment runoff during extreme cyclone events (described below). The probability constraint is implemented through a penalty term in the objective function, where MarProb predicts the probability that each feature contained in the proposed MPCA network will be represented in the protected area system with a given level of certainty across all features (Watts et al., 2021).

For each scenario, we generated 100 solutions, each with a different spatial configuration. To identify high‐priority areas for protection, we identified the best solution (i.e., the one with the lowest objective function score) and selection frequency (i.e., number of times a planning unit was selected across the 100 solutions) (Ball et al., 2009). We compared outputs between scenarios and created maps of the selection frequency of planning units and planning units selected in the best solution to evaluate the priority areas selected for protection with and without the inclusion of cyclone sediment runoff risk. To explore trade‐offs in planning MPCAs as an EbA approach that incorporates cyclone risk with traditional marine spatial planning approaches, we compared the overall cost to fishers and conservation feature targets achieved from the best solution for both planning scenarios.

### Conservation features and targets

Coral reefs, mangroves, and seagrass were used as conservation feature target ecosystems because they support biodiversity and subsistence fisheries provisions in coastal communities in the region (Andradi‐Brown et al., 2022; Mangubhai et al., 2019) (Table 1; Figure 1). Sea turtle feeding grounds were also included as a key conservation feature in the region because they support a priority endangered species that are affected by sediment runoff degrading benthic habitats, such as coral reefs and seagrass (Andradi‐Brown et al., 2022; Mangubhai et al., 2019) (Table 1; Figure 1).

### Costs to fishers

Spatially explicit information on fishing effort was not available for the GSR, and obtaining such fine‐scale data in the field would be costly. As such, we applied an already established method for data‐poor regions in which coastal population is used as a relative proxy to estimate fishing pressure on coastal and marine ecosystems (Ban et al., 2009; Klein et al., 2012). Coastal population counts were derived for major towns and cities adjacent to the GSR from Fijian census population information (accessed from www.citpopulation.de/en/fiji/admin) and used in a kernel density model of expected counts in ArcGIS to generate a cost raster (ESRI, 2011; Silverman, 1986) (see Figure 1 and Appendix S7 for towns and cities used).

### Sediment runoff threat map

We developed a land–sea modeling framework composed of 2 components: a sediment runoff model based on historic rainfall during extreme cyclone events and a coastal sediment dispersion model to couple with a predictive coral cover model. Inputs and outputs for the sediment runoff and dispersion model were processed using a combination of ArcGIS (ESRI, 2022) and the R programming language (R Core Team, 2022). As predictions of future cyclone dynamics and rainfall in Fiji remain complex (Dunstan et al., 2018; Knutson et al., 2010), we used historical cyclone patterns and rainfall events during extreme cyclone event periods as a proxy for the future threat under climate change (Levy & Ban, 2013; Mumby et al., 2014; Wolff et al., 2016).

To model sediment runoff from watersheds into coastal catchments across the GSR, we used the open‐source Nonpoint Source Pollution and Erosion Comparison Tool (OpenNSPECT) (Eslinger et al., 2012) in MapWindow GIS (https://www.mapwindow.org/). OpenNSPECT combines data on elevation, slope, soils, precipitation, rainfall erosivity, and land‐cover classifications to derive estimates of runoff, erosion and pollutant sources (nitrogen, phosphorous, and suspended solids), and accumulation in stream and river networks (Eslinger et al., 2012; Maina et al., 2012; Nam et al., 2003).

Watershed boundaries for Vanua Levu and Viti Levu Islands in the GSR were delineated based on small subcatchments on a Shuttle Radar Topographic Mission (SRTM) ‐derived Digital Elevation Model (DEM) with 90‐m spatial resolution in OpenNSPECT (Farr et al., 2007; NOAA, 2014a). Coastal river mouth drainage pour points for watersheds were determined based on the OpenNSPECT watershed delineations and validated from Google Earth imagery. The data inputs used in OpenNSPECT are described in more detail in Appendices S1 and S2.

### Modeling runoff

We used OpenNSPECT (Eslinger et al., 2012) to model total accumulated sediment runoff at river pour points throughout watersheds adjacent to the GSR during extreme cyclone events. OpenNSPECT for storm events utilizes a modified universal soil loss equation (MUSLE) (NOAA, 2014a, 2014b) as follows:

(4) $$S=a\times \;{\left(Q\times q_{p}\right)}^{b}\times K\times C\times P\times \mathrm{LS},$$

where *S* is the sediment yield from a storm event, *Q* is the storm event runoff volume, *q*_p_ is the peak runoff rate, *K* is the soil erodibility factor, *C* is a cover management factor that varies for each land‐use type based on literature for similar land‐cover classes (see Appendix S2), *P* is the supporting practices factor, LS is the slope length factor derived from the DEM, which adjusts erosion rates based on the steepness of the topography (Renard et al., 1997), and *a* and *b* are calibrated coefficients (NOAA, 2014b). We used modified coefficients calibrated to wet conditions persistent in Hawaii as a proxy for conditions in Fiji (NOAA, 2014b). Although OpenNSPECT produces numerical output values, we interpreted them as relative to each other rather than as actual absolute estimates of sediment loads (similar to Gibbs & West, 2019).

### Sediment dispersion model

We used a sediment dispersion model described in Brown et al. (2017) that measures the influence of different sediment sources on ocean turbidity or TSS at different distances from river mouth source pour points. The model describes the declining influence of source sediment loads (*j*) on ocean turbidity or TSS at an ocean site in the GSR seascape (*z_i_*_,_ *_j_*) with a power function:

(5) $$z_{i,j}=\beta_{j}d_{i,j}^{\alpha }$$

where β*_j_* is the influence of source sediment loads *j* on ocean turbidity or TSS from a distance of zero (i.e., river mouth pour point), α*_j_* is a scaling parameter that controls the dispersion of sediment, and d_i,j_ is a matrix of distances in kilometers from all ocean sites in the GSR seascape to river mouth sources. We expected α*_j_* to be negative if turbidity or TSS declined at greater distances from river mouth sources. We used a dispersion parameter of −2.3 to represent the northern coast of Fiji, following Brown et al. (2017). This is a relatively simple sediment dispersion model that is straightforward and repeatable for agencies working in data‐limited regions that do not have fine‐resolution data on coastal hydrodynamics, such as in many regions in Fiji. However, because this model does not include hydrodynamic or oceanographic processes, such as wind, bathymetry, and currents, which may alter the risk of sediment runoff from extreme cyclone events on coral reefs, this model limitation should be considered during on‐ground decision‐making.

### Coral reef surveys

Coral reef benthic surveys were performed in 2019 along three 50‐m transects at 72 sites in the GSR (Andradi‐Brown et al., 2022). Benthic habitat cover was surveyed at 0.5‐m intervals along each transect with a point‐intercept method, resulting in 100 benthic points recorded per transect (Andradi‐Brown et al., 2022). Benthic substrate types recorded included bare substrate, crustose coralline algae, hard coral, macroalgae, rubble, sand, soft coral, sponge, turf algae, and other invertebrate groups (Andradi‐Brown et al., 2022).

### Predictive probability map of hard coral cover

**FIGURE 2:**
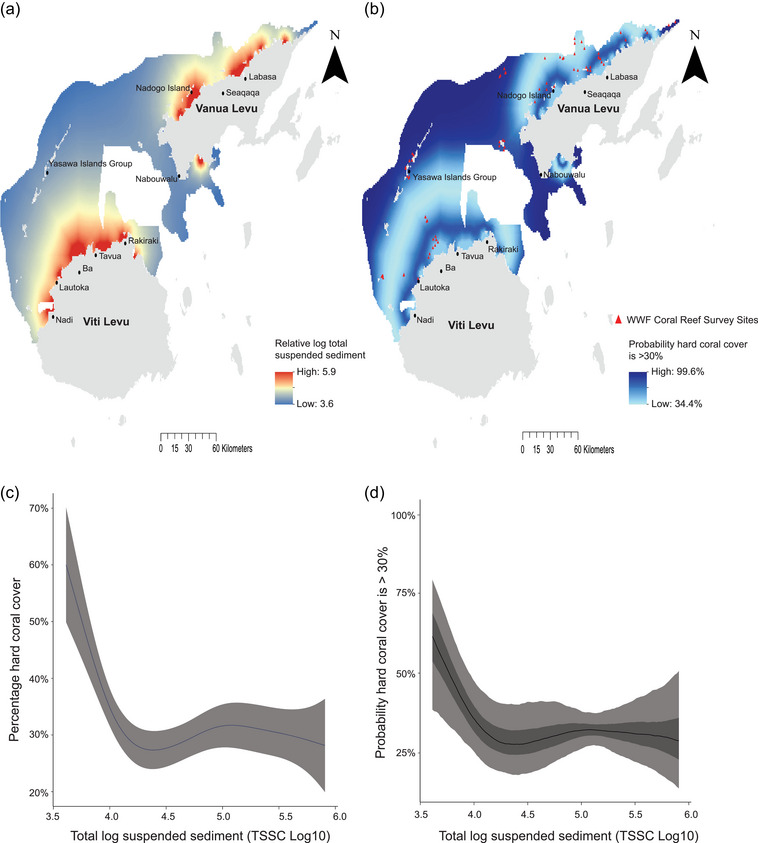
(a) Modeled relative total suspended sediment across the Great Sea Reef (GSR), Fiji, (b) probability of hard coral cover being >30% across the GSR when exposed to sedimentation during extreme cyclone events (WWF, World Wildlife Fund), (c) predicted response of hard coral cover (gray shading, SE) to relative total log suspended sediments during extreme cyclone events (82.6% deviance explained), and (d) posterior probability predictions of hard coral cover being >30% under different levels of relative total log suspended sediment (light gray shading, 95% probability intervals; dark gray shading, 50% probability intervals).

We used a binomial generalized additive model (GAM) to model the response of hard coral cover to TSS during extreme cyclone events in the GSR with the mgcv package in R (Wood, 2023). In the model, site was included as a random effect, and TSS was smoothed with a thin plate regression spline. Prior to running the model, TSS was log‐transformed to reduce skewness in the data. We confirmed there was no overdispersion in model residuals and identified no spatial autocorrelation in model residuals when checked with a semivariogram (Appendix S4). The fitted GAM model was used to predict hard coral cover across the entire GSR and then the probability of hard coral cover being >30% under sediment runoff levels during extreme cyclone events (Figure 2c,d). Predicted hard coral cover from the GAM was compared against the observed hard coral cover values to confirm the model's fit (Appendix S5).

A probability threshold of 30% hard coral cover was used to represent live hard coral cover in good condition in the GSR region. This value falls within the hard coral cover range considered an indicator of coral cover in good condition by AIMS (2021) and met the probabilistic biodiversity representation targets when running the MarProb cyclone risk scenario (see Appendix S3 for details). From the GAM model, we generated probability distributions for hard coral cover following the empirical Bayesian sampling method of Wood (2017) (Figure 2d). These were converted to a raster map of probability of hard coral cover being in good condition (hard coral cover >30%) (Figure 2b). The resulting coral probability layer was clipped to mapped coral reef areas by the Allen Coral Atlas (2022) to generate a probability layer of mapped coral reef areas with >30% hard coral cover in response to sediment runoff during extreme cyclone events in the GSR (Figure 2b).

## RESULTS

### Sediment runoff threat and hard coral cover probability

The greatest dispersion and highest relative concentrations of log TSS (range 3.6–5.9) were modeled around the coastal areas adjacent to the town of Ba along northern Viti Levu and around the coastal areas adjacent to the town of Labasa and Nadogo Island along northern Vanua Levu (Figure 2a). The probability of coral cover being in good condition (hard coral cover >30%) based on relative log TSS levels during extreme cyclone events was generally highest (>90%) further from the coastline of the 2 main islands of Viti Levu and Vanua Levu, where the relative total log suspended sediment was <4 or around 7 times lower than the maximum total log TSS (Figure 2a,b). An exception to this trend was in some inshore areas close to western, southwestern, and far northeastern Vanua Levu (Figure 2a,b). In other inshore areas, a relatively high probability (∼80%) that hard coral cover was in good condition was present where total log TSS was generally at moderately high levels (∼5) or around 2 times lower than the maximum total log TSS. Where relative log TSS was very high (>5.5) in inshore areas close to the coastline of the main islands, relatively low to moderate (≤56%) probability that coral was in good condition was typically present (Figure 2a,b). The lowest hard coral cover probability (∼34%) was in central GSR, where relative log TSS was moderate (∼4.5) or approximately 4 times lower than the maximum total log TSS (Figure 2a,b).

### Best solution spatial planning scenario comparisons

**FIGURE 3:**
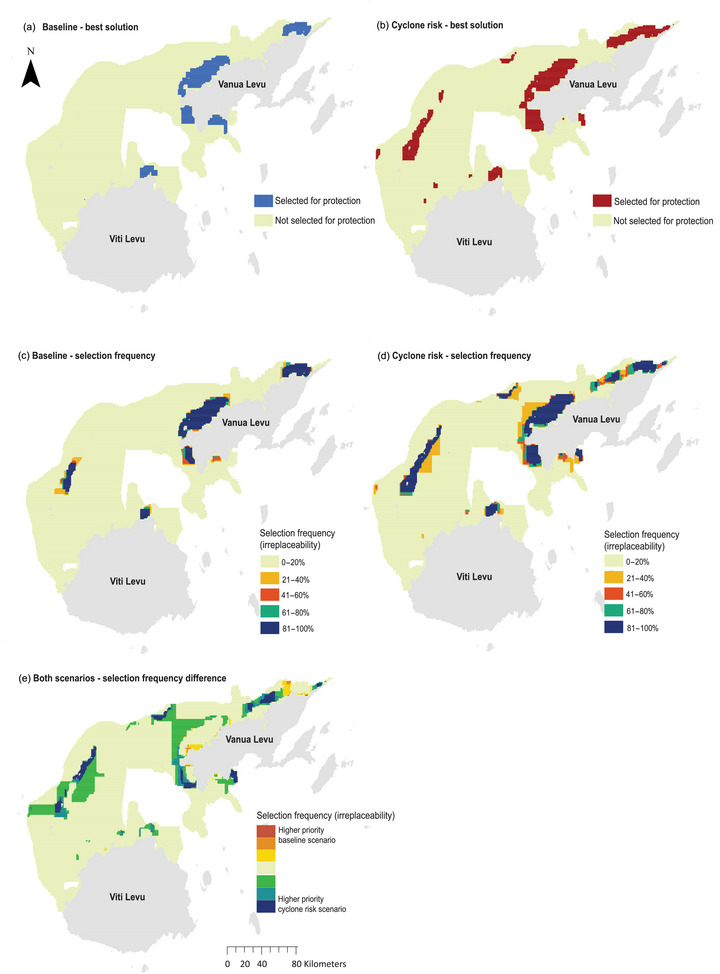
Spatial planning outputs for priority areas selected for protection for (a) baseline (no accounting for cyclone risk and hard coral condition) and (b) cyclone risk scenarios; selection frequency of areas for achieving conservation feature targets for the (c) baseline and (d) cyclone risk spatial scenarios; and (e) difference between the 2 scenarios.

Priority planning units selected for protection under the best solution for the baseline scenario (traditional Marxan) were largely concentrated around the northeast, northwest, west, and southwest of Vanua Levu, where there were lower fisher opportunity costs and a generally high concentration of all conservation features, including particularly large extents of sea turtle feeding grounds (Figures 1 & 3a; Appendix S7). Small concentrations of planning units were also selected around northern Viti Levu adjacent to the town of Rakiraki for similar reasons (Figure 3a). The cyclone risk scenario (MarProb) prioritized many similar areas for protection to those selected in the baseline scenario, including some locations with a relatively low probability of coral cover being in good condition (<40%) (Figures 3a,b). However, additional areas were selected for protection in the cyclone risk scenario in the offshore Yasawa Islands group; several small planning unit concentrations in the offshore boundary extent of the GSR and inshore north of Viti Levu; and some additional planning units selected in the northwest and northeast of Vanua Levu (Figure 3a,b). Small differences in the specific planning units selected for protection in southwestern Vanua Levu and near the town of Rakiraki were also observed (Figure 3a,b). The additional locations and planning units selected for protection in the cyclone risk scenario were generally located in areas with moderate to very high probability (>50%) of coral cover being in good condition (Figures 2b & 3b). Priority areas selected for protection in the cyclone risk scenario also included planning units that had high relative log TSS (>5.5) (Figures 2a & 3b).

When comparing trade‐offs in cost between the best solutions, the baseline scenario achieved protection targets across all conservation features for less than half the relative cost of the cyclone risk scenario (2407 and 5904 relative fisher opportunity loss cost, respectively) (Appendix S8). To achieve a 90% certainty that the coral reefs being protected in the cyclone risk scenario were in good condition, 5% more area in the GSR planning region was selected for priority protection (2751 km^2^, 13% total GSR area) across 1008 km^2^ of total conservation feature representation when compared with the baseline scenario (1657 km^2^, 8% total GSR area across 853 km^2^ of total conservation feature representation) (Appendix S8).

### Selection frequency spatial planning scenario comparisons

The baseline scenario identified 1485 km^2^ (7%) of marine and coastal area in the GSR consistently selected as a high priority for protection (>80% selection frequency), whereas the cyclone risk scenario identified 2156 km^2^ (10%) (Figure 3c,d). For the baseline scenario, there was little variation in the areas selected for protection, with 74% of the planning units that had a selection frequency of >20% considered a high priority for protection (>80% selection frequency) (Figure 3c). There were spatial consistencies in locations selected as a high priority for protection. Sixty‐three percent of the cyclone risk scenario area overlapped with the baseline scenario area, and, conversely, 92% of the baseline scenario area overlapped with the cyclone risk scenario area. Both scenarios consistently selected planning units in northwestern, northeastern, and western Vanua Levu, Yasawa Islands group, and inshore areas adjacent to the town of Rakiraki in northern Viti Levu for high‐priority protection (Figure 3c,d). A proportion of the high‐priority areas for the cyclone risk scenario comprised planning units with a low probability (<40%) of hard coral cover being in good condition (9%) or of no coral cover (16%); many of these were in areas also selected as a high priority in the baseline scenario because of low opportunity loss costs and presence of other conservation features or to increase the spatial compactness of the scenario outcome (Figures 2b & 3c,d).

There were several spatial differences in locations with high selection frequency in the cyclone risk scenario compared with the baseline scenario. These included additional areas in the Yasawa Islands group; southwest, west, and northeast Vanua Levu; and a concentration of planning units on the outer boundary of the GSR offshore from Vanua Levu (Figure 3e). Similar to the differences in locations selected for protection in the best solutions, these additional planning units in the cyclone risk scenario were commonly in areas with moderate to very high probability (generally >70%) of coral cover being in good condition (Figures 2b & 3e). Priority areas selected with high frequency in the cyclone risk scenario also included some planning units that had moderately high to high relative log TSS (>5–5.5) (Figures 2a & 3d,e).

## DISCUSSION

Incorporating information on cyclone threat risk in conservation and adaptation decision‐making is necessary to increase the socioeconomic resilience of communities to extreme cyclone events, given likely increases in the frequency of these events under climate change in many vulnerable PICTs (IPCC, 2022; Krauss & Osland, 2020). We developed a land–sea prioritization framework that expands on traditional threat‐avoidance planning approaches (e.g., Ban et al., 2012) by including ecological responses to a threat in the decision‐making process. By designing MPCA networks that included information on coral reef condition from sediment runoff during extreme cyclone events, we aimed to make the networks more resilient to ecological degradation due to extreme cyclone events. In doing so, we also reduced potential negative socioeconomic risks to local human communities dependent on these coral reefs for livelihoods and survival. By comparing trade‐offs between MPCA designs that include cyclone risk, fisher cost, and biodiversity representation with those that ignore cyclone threats, we found that including risk increases the cost and size of reserves but buffers against uncertainty of habitat condition. Our findings can inform EbA planning processes for the implementation of MPCAs in coastal regions threatened by climate change and can help tropical nations develop resilient 30×30 protected area systems.

Our models predicted some expected interactions between sediment level, distance from shore, and probability of coral reefs being in good condition. For instance, planning units the farthest from the shore of the 2 main islands generally had lower sediment levels and the greatest probability of good coral condition, and lower condition coral reefs were generally present in inshore areas with high relative sediment levels. Similar coral–sediment dynamic trends have been documented in many coral reefs around the world (Bejarano & Appeldoorn, 2013; Fabricius, 2005; Wenger et al., 2016). Conversely, some unexpected coral–sediment interactions were observed, where moderately high sediment levels predicted relatively high probabilities of good‐condition coral cover in some inshore areas. These results may be due to the presence of coral species that are more tolerant to higher turbidity levels in these areas (Guest et al., 2016; Loiola et al., 2019) or the presence of confounding threats that impact coral condition that we did not consider in our models, such as coral bleaching. Increasing evidence suggests the potential role of turbidity in reducing coral bleaching on some inshore coral reefs during periods of high thermal stress (Cacciapaglia & van Woesik, 2016; Guest et al., 2016; Morgan et al., 2017). We found that moderate sediment levels generally provided the lowest probability of coral cover being in good condition and hypothesize that less sediment‐tolerant species may comprise these areas or the interaction between turbidity and other threats is less pronounced. If our planning had also accounted for threats not directly related to distance from shore (e.g., coral bleaching), our resulting MPCA design may have avoided some mid‐shore areas that have moderately high sediment levels but high bleaching risk. More on‐ground surveying is required around turbid areas across the GSR to better understand coral–sediment dynamics in the region, along with further modeling to assess the influences of other threats on these interactions.

In the cyclone risk scenario, some areas with relatively high sediment loads were prioritized for protection because they had high amounts of ecosystem conservation features or a relatively good chance of having coral cover in good condition (e.g., inshore areas in northwest Vanua Levu), or both. Under traditional approaches to including threats in prioritizations for conservation or adaptation planning, areas composed of relatively high TSS levels would normally have been excluded from protection (Tulloch et al., 2015, 2016). Our approach considers the broad tolerance of hard corals to sediment runoff and provides a pathway for further research to consider coral species‐ or genera‐specific tolerances to differing sediment levels in the GSR (Jokiel et al., 2014; Jones et al., 2020). Our findings reinforce the need for threat‐based models used in adaptation planning to consider the ecological responses of targeted habitats or species to threats, as aiming to just avoid areas with high threat levels may not always provide the most significant socioecological benefit (Tulloch et al., 2015). Similarly, an alternative prioritization approach would be to protect coral reefs most at risk of being in bad condition under cyclone threat to increase the resilience of these areas to climate change (Game, McDonald‐Madden, et al., 2008). However, in the context of EbA and the expected increasing frequency of extreme cyclone events under climate change in many regions, choosing to protect areas with coral cover in good condition will likely provide more reliable socioecological adaptation outcomes (Beyer et al., 2018; Bloemendaal et al., 2022; Giffin et al., 2020).

Our findings demonstrate that it is significantly more costly to incorporate certainty that habitat with coral cover in good condition is present in MPCAs when exposed to sediment runoff from extreme cyclone events. Other terrestrial and marine studies also show that higher costs are generally required to achieve certainty of habitat condition in protected area planning, but magnitudes of costs vary depending on the stressor, probability targets, and distribution of potential threat probabilities (Klein et al., 2013; Powers et al., 2017; Witt & Hammill, 2018). Our cyclone risk scenario had a wide distribution of potential probabilities that coral habitat was in good condition, which meant that some coral reefs in relatively poor condition were also selected for protection to achieve other conservation feature protection targets and additional areas of good‐quality coral reef were prioritized for protection at an extra cost. Although the MPCA design of the cyclone risk scenario is larger and more expensive, a significant advantage of including habitat condition certainty in the context of climate adaptation is a greater confidence that human communities will receive their intended ecosystem services from MPCAs and increased resilience to climate events (Giffin et al., 2020; Russ et al., 2003; Tulloch et al., 2013). Future research could compare these findings with a prioritization approach that has a budget constraint for the cyclone risk scenario and determine how much area it selects at this level (Remme & Schröter, 2016; Watson et al., 2011). Incorporating habitat certainty makes decision‐making more robust and precautionary, and investing in MPCAs as an EbA approach is less risky for funders (Regan et al., 2005; Tulloch et al., 2013).

A large concentration of priority areas selected for protection across both scenarios were identified around Vanua Levu. Protecting large areas of inshore waters around Vanua Levu has potentially inequitable socioeconomic implications for coastal communities in that region (Andradi‐Brown et al., 2022; Halpern et al., 2013). Although the total opportunity loss costs are lower in our study around Vanua Levu compared with the bigger main island of Viti Levu when we used population as a proxy for cost, Vanua Levu communities may have a greater reliance on coastal ecosystems due to possibly fewer alternative protein sources or livelihoods (Dacks et al., 2018). Marxan and MarProb best solutions are composed of a definite number of planning scenario runs and do not represent all possible planning solutions to complex socioecological problems (Klein et al., 2013). Similarly, changes to the hard coral cover probability threshold may change the costs and priority areas selected for protection in the cyclone risk scenario. Our MPCA scenario outputs should be considered as a decision support tool to be used by planners in conjunction with up‐to‐date on‐ground knowledge from communities and managers to achieve desired socioecological objectives under specified funding limits (Watts et al., 2021).

We used the GSR as a case study to explore the use of our land–sea planning framework to assist adaptation planners in implementing MPCAs as an EbA tool under the threat of cyclones on coral reefs in marine and coastal areas at a regional scale. However, when planning and implementing MPCAs in Fiji, there are several other management variables that need to be considered, such as local tenure units, established governance frameworks and systems, local capacity, and community buy‐in (Jupiter & Egli, 2011; WCS, 2016). Marine and coastal areas in the GSR are divided into local customary management units recognized by Indigenous Fijian communities as qoliqoli (Andradi‐Brown et al., 2022). Our land–sea framework can help highlight which qoliqoli are important to prioritize EbA action in a region. However, as traditional fishing rights are recognized in qoliqoli and locally managed at this level (Andradi‐Brown et al., 2022; Dacks et al., 2018), further research should test the use of zoning prioritization tools to set conservation or fisheries targets for each individual qoliqoli to assist with feasible on‐ground implementation and management of MPCAs as an EbA action in the GSR (e.g., Weeks et al., 2010). Planning at these smaller local tenure units will also assist with providing more equitable cost and benefit outcomes for all communities across the GSR.

There are several limitations to our study. We used a relatively simple sediment runoff and dispersion model to generate a TSS threat layer. More sophisticated models that include in situ river gauge data or oceanographic processes (e.g., wind, bathymetry, and currents) may assist with finer‐scale threat‐risk analysis (Brown et al., 2017; Delevaux et al., 2018). Moreover, we used historical data on cyclones and rainfall as a surrogate for levels expected to influence sediment runoff during extreme cyclone events under climate change. Future research could focus on conducting on‐ground surveys of GSR turbidity levels to validate our models or use more complex climate models to predict cyclone frequency, intensity, and associated rainfall under climate change projections and the cumulative risk of both direct and indirect cyclone impacts on coral reefs and how incorporating this information may change priority protection outcomes (Bloemendaal et al., 2022; Brown et al., 2017; Parker et al., 2018). We used population as a relatively simple proxy for fisher opportunity cost due to a lack of available spatially explicit data (Andradi‐Brown et al., 2022). Local surveys could improve knowledge and data availability on fishing dependence, but this would likely be costly to implement at a regional scale such as the GSR (Andradi‐Brown et al., 2022). Future research could focus on finer‐scale plans that may have more socioeconomic data available (e.g., Gurney et al., 2015). We also suggest verification of sea turtle feeding ground data at a local scale before decision‐making. We used the best available data to explore the outcomes of incorporating cyclone risk in the spatial prioritization process of designing networks of MPCAs at a regional scale. Our findings can inform EbA planning in other coastal regions where the sustainable delivery of ecosystem services to communities through traditional MPCA design is threatened by climate change.

## Supporting information



Appendix S1 OpenNSPECT sediment run‐off model
Appendix S2. C‐Factors used in the Open‐NSPECT Sediment Run‐off Model
Appendix S3 Predictions of hard coral cover under sediment threat from cyclones
Appendix S4. Semivariogram of residuals for the model of hard coral cover and total suspended solids.
Appendix S5. Predicted hard coral cover from the model versus observed hard coral cover from the field surveys.
Appendix S6 Marxan and Marxan with Probability (MarProb) spatial planning analysis
Appendix S7. Relative fishers opportunity loss cost across the Great Sea Reef. Major cities and towns located adjacent to the GSR are displayed and populations of these locations are used to calculate fisher opportunity loss costs.
Appendix S8. Comparison of attributes of the baseline versus cyclone risk scenario using Marxan/MarProb best solution outputs
