# Supplementary material for: Incorporating cyclone risk in the design of marine protected and conserved areas as an ecosystem‐based adaptation approach
Source: Conserv Biol. 2025 Feb 25;39(4):e14437. doi: 10.1111/cobi.14437 (PMC12309639; doi:10.1111/cobi.14437)
Supplement: Supplementary file 1 — Appendix S1 OpenNSPECT sediment run‐off model Appendix S2. C‐Factors used in the Open‐NSPECT Sediment Run‐off Model Appendix S3 Predictions of hard coral cover under sediment threat from cyclones Appendix S4. Semivariogram of residuals for the model of hard coral cover and total suspended solids. Appendix S5. Predicted hard coral cover from the model versus observed hard coral cover from the field surveys. Appendix S6 Marxan and Marxan with Probability (MarProb) spatial planning analysis Appendix S7. Relative fishers opportunity loss cost across the Great Sea Reef. Major cities and towns located adjacent to the GSR are displayed and populations of these locations are used to calculate fisher opportunity loss costs. Appendix S8. Comparison of attributes of the baseline versus cyclone risk scenario using Marxan/MarProb best solution outputs [file COBI-39-e14437-s001.docx]

**Incorporating cyclone risk in the design of marine protected and conserved areas as an ecosystem-based adaptation approach – Supplementary Material**

**Appendix S1**

**OpenNSPECT sediment run-off model**

*Soil data*

Two soil variables were used in the sediment run-off model: (1) hydrologic soil group that was classified into groups A, B, C, D to indicate the minimum rate of infiltration obtained for bare soil after prolonged wetting (Nam et al., 2003), and (2) soil erodibility (K-Factor) that described the soil's susceptibility to erosion by rain, as a function of sand, silt, clay and organic carbon concentration (Tulloch et al., 2016). The hydrologic soil groups for the GSR catchment were obtained from the Global Hydrologic Soil Groups (HYSOGs250m) raster and categorised into groups A, B, C and D. The soil erodibility K-Factors were derived from the Harmonized World Soil Database Version 1.2 raster (FAO/IIASA/ISRIC/ISS-CAS/JRC, 2009) and were calculated as an average for each soil raster pixel as per Maina et al. (2012) and Tulloch et al. (2016).

*Rainfall data*

Raw daily rainfall data was obtained from the Fiji Bureau of Meteorology between 1970-2018 at 35 weather stations across the Fijian Archipelago (Fiji Met, 2020). This raw rainfall data was subset to cyclone event periods based on cyclone tracks that occurred within 300 km of Suva between 1969-2018 obtained from the Australian Bureau of Meteorology (BOM, 2020). For each weather station, the maximum amount of rainfall (mm) during a cyclone event in the cyclone event period was retained. The cyclone event that had the highest maximum rainfall at each weather station was used as a proxy for rainfall during extreme cyclone events. This rainfall data was interpolated using the Inverse Distance Weighting (IDW) method in ArcMap (ESRI, 2007) and a moderate weighting value (3) was used to generate a spatial precipitation raster of maximum rainfall during extreme cyclonic events across the land catchments of Viti Levu and Vanua Levu associated with the GSR. The IDW method was chosen as it is a relatively simple and frequently used method for interpolating precipitation data (Yang et al., 2015). The specified rainfall type used in the OpenNSPECT analysis was Type I, which is consistent with Pacific maritime climates (NOAA, 2014).

*Land-use land-cover (LULC) data*

Land-use land-cover (LULC) classification data was obtained from the Pacific Catastrophe Risk Assessment and Financing Initiative (PCRAFI, 2020) and was reclassified as a raster based on 19 land-use and cover classes (Appendix S1). Each LULC delineation was assigned a cover management factor that indicates the effect of vegetation on soil erosion rates (Appendix S1; NOAA, 2014; Renard et al. 1997).

**Appendix S2.** C-Factors used in the Open-NSPECT Sediment Run-off Model

| Land class | C Factor | SCS  CCAP CN-A | SCS CCAP CN-B | SCS CCAP CN-C | SCS CCAP CN-D | Reference |
| --- | --- | --- | --- | --- | --- | --- |
| Barren Land | 1 | 0.7700 | 0.8600 | 0.9100 | 0.7000 | Tulloch et al., 2016 |
| Cassava | 0.4 | 0.6700 | 0.7800 | 0.8500 | 0.8900 | Delevaux et al., 2018 |
| Coconut Crops, Forest, Plantations | 0.2 | 0.6700 | 0.7800 | 0.8500 | 0.8900 | Delevaux et al., 2018 |
| Cultivated Land | 0.2 | 0.6700 | 0.7800 | 0.8500 | 0.8900 | Tulloch et al., 2016 |
| Forest | 0.002 | 0.3000 | 0.5500 | 0.7000 | 0.7700 | Delevaux et al., 2018 |
| Grazing Land | 0.075 | 0.3900 | 0.6100 | 0.7400 | 0.8000 | Silburn, 2011 |
| Mixed Crops | 0.2 | 0.6700 | 0.7800 | 0.8500 | 0.8900 | Tulloch et al. 2016 |
| Oil Palm | 0.25 | 0.6700 | 0.7800 | 0.8500 | 0.8900 | Average of oil palm categories from Tulloch et al. 2016 |
| Open Land/Grassland | 0.012 | 0.300 | 0.5800 | 0.7100 | 0.7800 | Delevaux et al. 2018 |
| Orchards | 0.2 | 0.6700 | 0.7800 | 0.8500 | 0.8900 | Given same value as cultivated land from Tulloch et al. 2016 |
| Other | 0.2 | 0.6700 | 0.7800 | 0.8500 | 0.8900 | Given most common value |
| Rice | 0.2 | 0.6700 | 0.7800 | 0.8500 | 0.8900 | Delevaux et al. 2018 |
| Scattered Coconut Plan | 0.2 | 0.6700 | 0.7800 | 0.8500 | 0.8900 | Delevaux et al. 2018 |
| Scattered Forest | 0.2 | 0.3000 | 0.5500 | 0.7000 | 0.7700 | Average of patchy forest cover Tulloch et al. 2016 |
| Settlement | 0.2 | 0.7700 | 0.8500 | 0.9000 | 0.9200 | Delevaux et al. 2018 |
| Shrubs | 0.008 | 0.3000 | 0.4800 | 0.6500 | 0.7300 | Delevaux et al. 2018 |
| Sugarcane | 0.2 | 0.3900 | 0.6100 | 0.7400 | 0.8000 | Given same as cultivated land from Tulloch et al. 2016 |
| Water | 0 | 0 | 0 | 0 | 0 | Tulloch et al. 2016 |
| Wetland/Mangroves | 0.0015 | 0 | 0 | 0 | 0 | Average between wetlands and mangroves from Delevaux et al. 2018 |

*Watershed delineation*

We used a resolution of 500m to allow processing across Fiji. Within OpenNSPECT, we tested both medium and small watersheds and decided to use small watersheds as this scale of delineation provided the greatest detail in terms of pour point accuracy and validation against satellite maps.

**Appendix S3**

**Predictions of hard coral cover under sediment threat from cyclones**

We conducted sensitivity analysis to determine an appropriate threshold of hard coral cover of > 30% under sediment run-off during extreme cyclone events, which is consistent with the range of >30% – 50% being indicators of good condition coral cover (AIMS, 2021), and met the probabilistic biodiversity representation targets when running the MarProb cyclone risk scenario. Although Andradi-Brown et al. (2022) indicates that 35% coral cover is closer to the current average within the GSR, we could not meet probabilistic biodiversity representation targets when the probability that hard coral was set at > 35% in the MarProb cyclone risk scenario analysis. This may be a limitation of our data and coral-sediment models or a representation that certainty of high levels of hard coral cover (> 35%) will be challenging to obtain through protection under sediment run-off during extreme cyclone events.

*Generalised Additive Model (GAM) outputs*


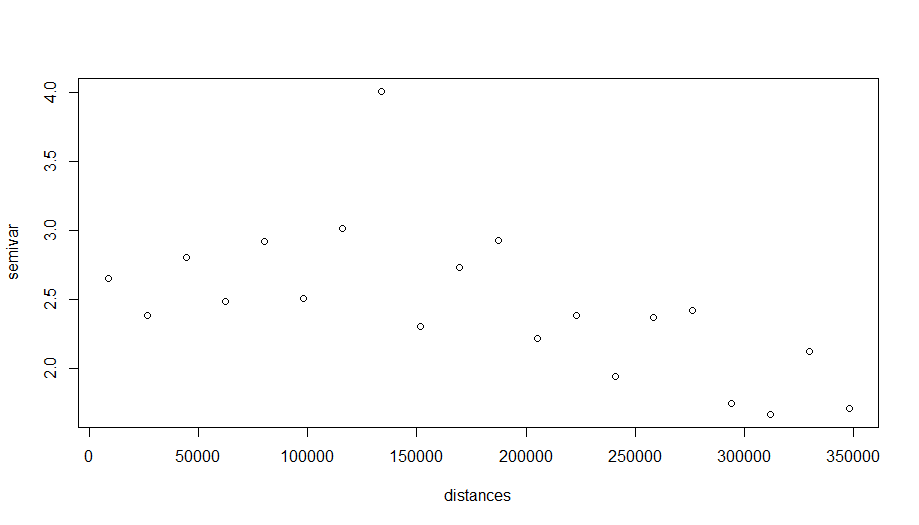


**Appendix S4.** Semivariogram of residuals for the model of hard coral cover and total suspended solids.

**
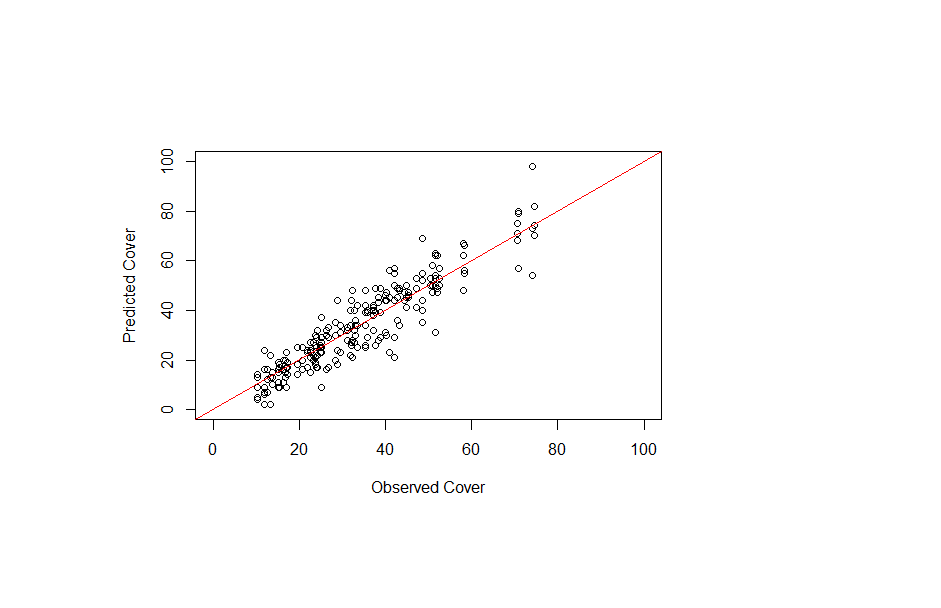
**

**Appendix S5.** Predicted hard coral cover from the model versus observed hard coral cover from the field surveys.

**Appendix S6**

**Marxan and Marxan with Probability (MarProb) spatial planning analysis**

We used calibrations to determine a realistic level of clumping for the priority areas for protection by varying the boundary length modifier (BLM) parameter for the baseline scenario in Marxan and the cyclone risk scenario in MarProb and chose a blm of 0.05. We also used calibrations to determine a species penalty factor (spf) of 10 for all conservation features in the baseline scenario in Marxan and the cyclone risk scenario in MarProb. A certainty areal representation target (ptarget2D) was calibrated for hard coral in the cyclone risk scenario in MarProb and given a ptarget2D of 0.9. All conservation features that were not coral (i.e., mangrove, sea turtle feeding grounds and seagrass) were given a constant ptarget2D of 0.999.

*Cost layer*

We used the kernel density tool in ArcMap (ESRI, 2011) of expected population counts with a search radius set to 50km and an output cell size of 1km to match the planning unit raster cell size of 1km. The KernelDensity tool (ESRI, 2011) combines distance and density by creating a smooth curved surface fitted over each point where the surface value is highest at the location of the point and diminishes based on a quadratic kernel function from that point (Silverman, 1986).

**
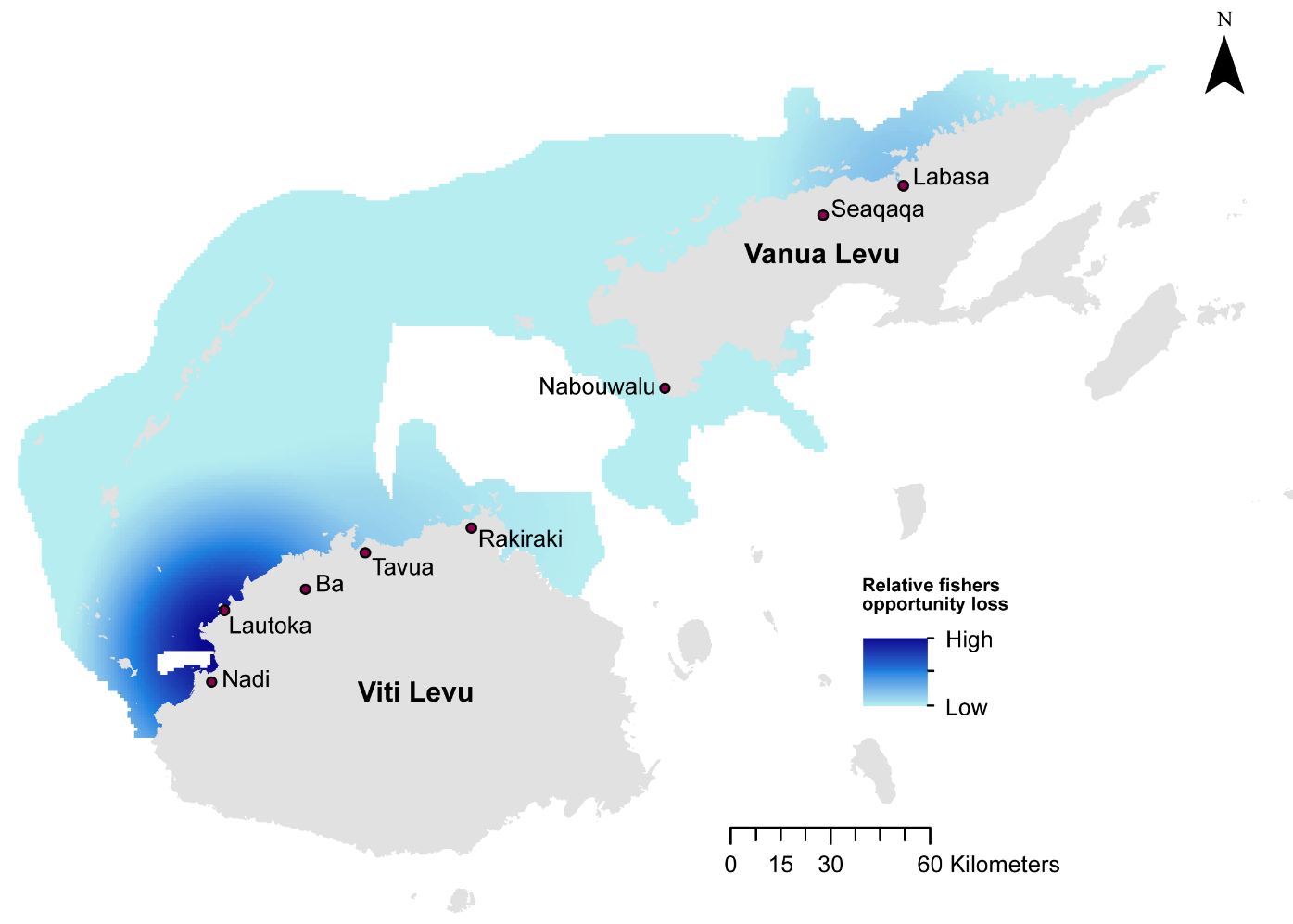
**

**Appendix S7.** Relative fishers opportunity loss cost across the Great Sea Reef. Major cities and towns located adjacent to the GSR are displayed and populations of these locations are used to calculate fisher opportunity loss costs.

*Spatial planning outputs*

**Appendix S8.** Comparison of attributes of the baseline versus cyclone risk scenario using Marxan/MarProb best solution outputs

| **Scenario** | **Conservation feature representation (area m^2^)** | **Number of selected planning units (occurrences)** | **Relative fisher opportunity loss cost value** |
| --- | --- | --- | --- |
| **Baseline (Marxan)**    Seagrass  Mangrove  Sea turtle feed grounds  Coral  Total | 109,716,907  85,734,784  414,244,151  243,003,657  852,699,499 | 1,058  356  499  1,399  1,657 (duplicates removed) | 2,407 |
| **Cyclone risk (MarProb)**  Seagrass  Mangrove  Sea turtle feed grounds  Coral  Total | 118,754,835  86,290,597  423,279,065  379,924,568  1,008,249,065 | 1,549  415  514  2,259  2,751 (duplicates removed) | 5,904 |

**References**

AIMS, 2021. Annual Summary Report of Coral Reef Condition 2020/2021, Australian Institute of Marine Science. Townsville, Australia.

Andradi-Brown, D.A., Veverka, L., Free, B., Ralifo, A., Areki, F., National Geography Society, Washington, D.C, U., Ocean Conservation, World Wildlife Fund, Washington, D.C, U., WWF - Pacific Programme, Suva, F., 2022. Status and trends of coral reefs and associated coastal habitats in Fĳi’s Great Sea Reef 96–150. https://doi.org/10.6084/m9.figshare.13228910

Delevaux, J.M.S., Jupiter, S.D., Stamoulis, K.A., Bremer, L.L., Wenger, A.S., Dacks, R., Garrod, P., Falinski, K.A., Ticktin, T., 2018. Scenario planning with linked land-sea models inform where forest conservation actions will promote coral reef resilience. Sci. Rep. 8, 1–21. https://doi.org/10.1038/s41598-018-29951-0

ESRI, 2011. How Kernel Density Works. https://webhelp.esri.com/arcgisdesktop/9.3/index.cfm?TopicName=How Kernel Density works

FAO/IIASA/ISRIC/ISS-CAS/JRC, 2009. Harmonized World Soil Database (version 1.1). FAO, Rome, Italy and IIASA, Laxenburg, Austria.

Nam, P.T., Yang, D., Kanae, S., Oki, T., Musike, K., 2003. Global soil loss estimate using RUSLE model: the use of global spatial datasets on estimating erosive parameters. Geoinformatica 14, 49-53.

NOAA, 2014. User’s Manual for OpenNSPECT, Version 1.2. Charleston, South Carolina.

Renard, KG, Foster, GR, Weesies, GA, McCool, D, Yoder, A., 1997. Predicting soil erosion by water: a guide to conservation planning with the Revised Universal Soil Loss Equation (RUSLE). Agriculture Handbook (Washington). https://www.ars.usda.gov/arsuserfiles/64080530/rusle/ah_703.pdf

Silburn, D.M., 2011. Hillslope runoff and erosion on duplex soils in grazing lands in semi-arid central Queensland. III. USLE erodibility (K factors) and cover-soil loss relationships. https://doi.org/10.1071/SR09070

Silverman, B.W., 1986. Density estimation for statistics and data analysis. Chapman Hall, New York. https://ned.ipac.caltech.edu/level5/March02/Silverman/paper.pdf

Tulloch, V.J.D., Brown, C.J., Possingham, H.P., Jupiter, S.D., Maina, J.M., Klein, C., 2016. Improving conservation outcomes for coral reefs affected by future oil palm development in Papua New Guinea. Biol. Conserv. 203, 43–54. https://doi.org/10.1016/j.biocon.2016.08.013

Yang, X., Xie, X., Liu, D.L., Ji, F., Wang, L., 2015. Spatial Interpolation of Daily Rainfall Data for Local Climate Impact Assessment over Greater Sydney Region. Adv. Meteorol. 2015. https://doi.org/10.1155/2015/563629
